# Validation of satellite-based sea surface temperature products against in situ observations off the western coast of Sumatra

**DOI:** 10.1038/s41598-021-04156-0

**Published:** 2022-01-07

**Authors:** Qoosaku Moteki

**Affiliations:** Dynamic Coupling of Ocean-Atmosphere-Land Research Program (DCOP), Japan Agency for Marine-Earth Science and Technology (JAMSTEC), 2-15 Natsushima-cho, Yokosuka City, Kanagawa 237-0061 Japan

**Keywords:** Physical oceanography, Atmospheric dynamics

## Abstract

This study validated the sea surface temperature (SST) datasets from the Group for High-Resolution SST Multi Product Ensemble (GMPE), National Oceanic and Atmospheric Administration (NOAA) Optimal Interpolation (OI) SST version 2 and 2.1 (OIv2 and OIv2.1), and Estimating the Circulation and Climate of the Ocean, Phase II (ECCO2) in the area off the western coast of Sumatra against in situ observations. Furthermore, the root mean square differences (RMSDs) of OIv2, OIv2.1, and ECCO2 were investigated with respect to GMPE, whose small RMSD < 0.2 K against in situ observations confirmed its suitability as a reference. Although OIv2 showed a large RMSD (1–1.5 K) with a significant negative bias, OIv2.1 (RMSD < 0.4 K) improved remarkably. In the average SST distributions for December 2017, the differences among the 4 datasets were significant in the areas off the western coast of Sumatra, along the southern coast of Java, and in the Indonesian inland sea. These results were consistent with the ensemble spread distribution obtained with GMPE. The large RMSDs of OIv2 corresponded to high clouds, and it was suggested that the change in the satellites used for SST estimation contributed to the improvement in OIv2.1.

## Introduction

Years of the Maritime Continent (YMC^1^) is a multiyear (from 2015 to present) international program with participants from more than 15 countries. Its goal is to improve the understanding of the local oceanic and atmospheric multiscale variability of the Indo-Pacific maritime continent (MC), and the initial results have been published^2–9^.

Gridded gap-free objective analyses of sea surface temperature (SST), which are generally called Level 4 (L4) data, are frequently used in such studies. In addition, L4 SST datasets are conventionally used as boundary conditions in atmospheric reanalysis, climate monitoring, and weather forecasting and are widely used in studies of phenomena involving air-sea interactions, such as the Madden–Julian oscillation (MJO^10^) and boreal summer intraseasonal oscillation (BSISO^11^). Understanding the characteristics of and differences in the various L4 SST datasets in the vicinity of the MC is very important as a premise for summarizing the various results of YMC.

Many L4 SST datasets have distinct characteristics depending on the assimilation methods adopted, the choice of satellites, the number of in situ observations, and the temporal-spatial resolution, and the differences in values and distributions among datasets can be misleading in discussions summarizing many YMC results. In particular, the validation of L4 SST in shallow coastal sea areas is difficult because there are very few in situ SST observations with buoys and Argo floats, although uncertainties in satellite estimations greatly increase near coasts and marginal seas with small islands.

Therefore, we need to understand the characteristics of L4 SST datasets in a specific region for a specific period apart from the validation, which is generally performed as a global average or over a longer period by the dataset providers. The L4 SST Quality Monitor (L4-SQUAM) system is aimed at cross-comparisons of various L4 products and monitors the quality of various L4 products globally in near-real time by comparison with the Group for High-Resolution SST Multi Product Ensemble (GMPE^12,13^). GMPE is provided on a daily basis by the UK Met Office, taking 16 L4 products as inputs and producing an ensemble median and standard deviation with a 1/4° grid^13^. With respect to near-surface Argo float observations, GMPE is globally more reasonable than any of the contributing SST products, with a standard deviation of 0.40 K.

However, confirming the suitability of GMPE is necessary because the observation frequency of in situ SST by Argo is approximately once every 10 days, and the number of Argo floats is very small in shallow coastal sea areas. In addition, GMPE has the disadvantages of providing a short dataset period (from 16 Sep. 2017 to present) and no anomaly values from the climatological mean. GMPE cannot be used in YMC studies on phenomena prior to Sep. 2017, and many YMC studies require the SST anomaly dataset. To compensate for these disadvantages of GMPE, it is necessary to investigate how quantitatively consistent the other datasets with the long-term stable operation are.

This study verifies the L4 SST datasets, including GMPE, in the area off the western coast of Sumatra against in situ water temperature values at 10 m depth obtained from the Sumatra buoy (5° S, 100° E) during the YMC period and conductivity-temperature-depth (CTD) profiles obtained by the Research Vessel (R/V) Mirai (fixed at 4.24° S, 101.52° E for the period of 5 Dec. 2017–1 Jan. 2018) during the cruise named MR17-08^14^. The National Oceanic and Atmospheric Administration (NOAA) Optimal Interpolation (OI) SST version 2 (OIv2) is one of the most popular L4 datasets and has been widely used in many studies. On 1 Apr. 2020, version 2.1 of NOAA OI SST (OIv2.1) was published. Although there recently are many other modern SST datasets that substantially outperform even OIv2.1, many of the air-sea interaction researches often require the SST anomaly distribution from the climatological mean and analyses using the same conditions and indices as in previous studies. That is the primary reason for the validation of OIv2 and OIv2.1 that have the longest homogeneous record in this study.

Furthermore, we chose the Estimating the Circulation and Climate of the Ocean, Phase II (ECCO2), as a verification target product based on a three-dimensional (3D) ocean model. In 3D ocean objective analysis, such as ECCO2, SST is not only constrained by satellite and in situ observations but also affected by the dynamic ocean velocity field. The reason for choosing ECCO2 is that it is updated relatively more quickly and archived for a longer period among the ocean objective analysis datasets.

The purpose of the present study is to verify the 4 distinct L4 SST datasets (GMPE, OIv2, OIv2.1, and ECCO2) against the YMC in-situ observations of Sumatra buoy and CTD profiles. This study summarizes the characteristics of the four L4 SST datasets, which are estimated as so-called “bulk” SST (the temperature of the mixed water column in the layer of 1–10 m depth) off the western coast of Sumatra during the YMC period and shows a premise for the previous and ongoing studies of YMC.

## Results

### Validation for the years 2017–2019

As in the average for the entire globe, GMPE is the most reasonable L4 SST according to the validation against the reference of Argo floats with a standard deviation of 0.40 K. Through the validations in this study, RMSD less than 0.4 K, which is the global mean standard deviation of GMPE, is interpreted as "sufficiently small” RMSD.

**Figure 1:**
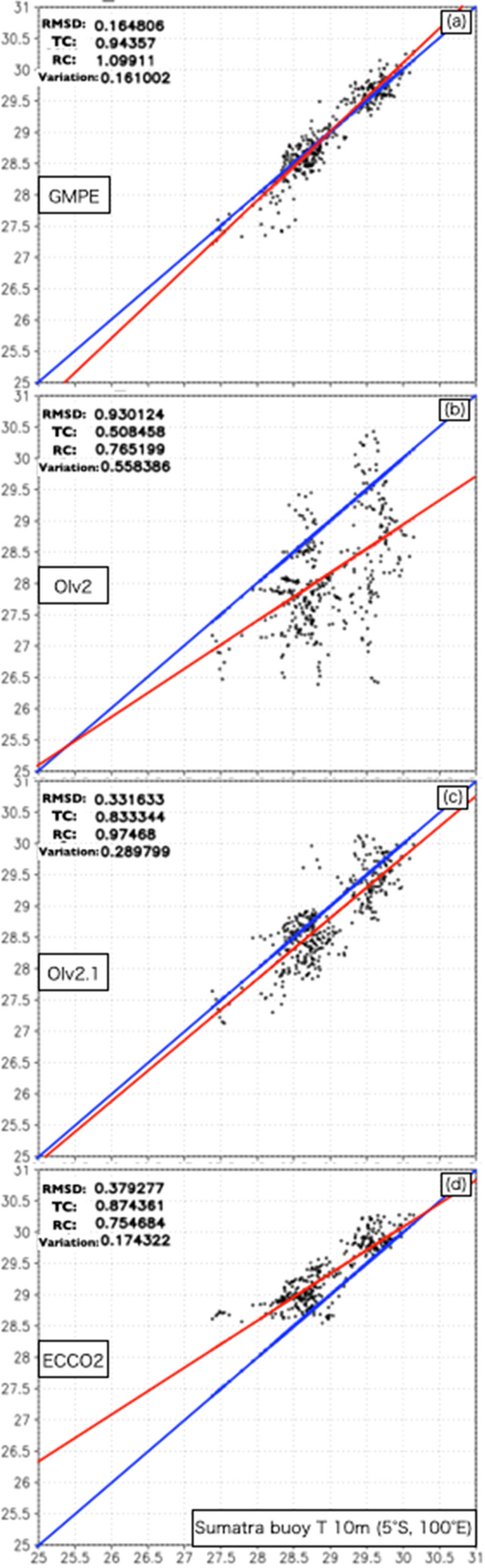
Scatter plots for (**a**) GMPE, (**b)** OIv2, (**c**) OIv2.1, and (**d**) ECCO2 at 5° S, 100° E for the period from 6 Dec. 2017–30 Nov. 2018 with respect to the Sumatra buoy. The temporally averaged RMSD, TC, RC, and variance are shown in the top left corner of each panel. The regression line and the line with a slope of 1 are shown by the red and blue lines, respectively. The black box shows the area for averaging RSMD in Fig. 2.

First of all, we validate GMPE against the reference of Sumatra buoy and CTD from the MR17-08 cruise to confirm GMPE quality in the area off the western coast of Sumatra. Figure 1 shows scatter plots between SST datasets, and temporally averaged indices are summarized in Table 1. First, the GMPE and the Sumatra buoy for the period 6 Dec. 2017–30 Nov. 2018 are compared in Fig. 1a. GMPE is very consistent with the in situ observations (a temporally averaged RMSD of 0.16 K, time correlation (TC) of 0.94, regression coefficient (RC) of 1.1, and variance of 0.16 K). Near the shallow coastal sea (approximately 1000 m depth), short-term (within a week) fluctuations in the ocean current direction due to coastal tides and eddies were observed^14^. As shown in Table 1, the TC and RC values against the Mirai CTD were smaller than those in the open ocean at the Sumatra buoy (more than 5500 m depth). These values show that the use of GMPE as a reference is reasonable in the area off the western coast of Sumatra because the RMSD of GMPE is sufficiently smaller than the global mean standard deviation of 0.4 K according to validation against the reference of Argo floats. The indices of OIv2 (Fig. 1b) off the western coast of Sumatra are the worst among the selected L4 datasets. The large RMSD is due to a large negative bias. Although OIv2.1 (Fig. 1c) shows a negative bias, it is significantly improved from OIv2. However, the TC value is relatively low, and the variance is still large in the shallow coastal sea. The RMSD of ECCO2 (Fig. 1d) is due to a positive bias, and ECCO2 shows an overestimation trend for low SSTs less than 29 °C. Notably, the variance is quite small, and the TC value is higher than that of OIv2.1.

**Table 1:** The root mean squared difference (RMSD), time correlation (TC), regression coefficient (RC), and variance of GMPE, OIv2, OIv2.1, and ECCO2 with respect to the Sumatra buoy (5° S, 100° E) averaged for 6 Dec. 2017–30 Nov. 2018 [R/V Mirai CTD (4° S, 101° E) averaged for 6–31 Dec. 2017].

L4 SST dataset	RMSD (K)	TC	RC	Variance (K)
GMPE	0.16 [0.07]	0.94 [0.60]	1.10 [0.46]	0.16 [0.05]
OIv2	0.93 [1.43]	0.51 [0.46]	0.51 [2.16]	0.56 [0.40]
OIv2.1	0.33 [0.42]	0.83 [0.22]	0.83 [0.83]	0.29 [0.39]
ECCO2	0.37 [0.49]	0.87 [0.35]	0.75 [0.17]	0.17 [0.04]

**Figure 2:**
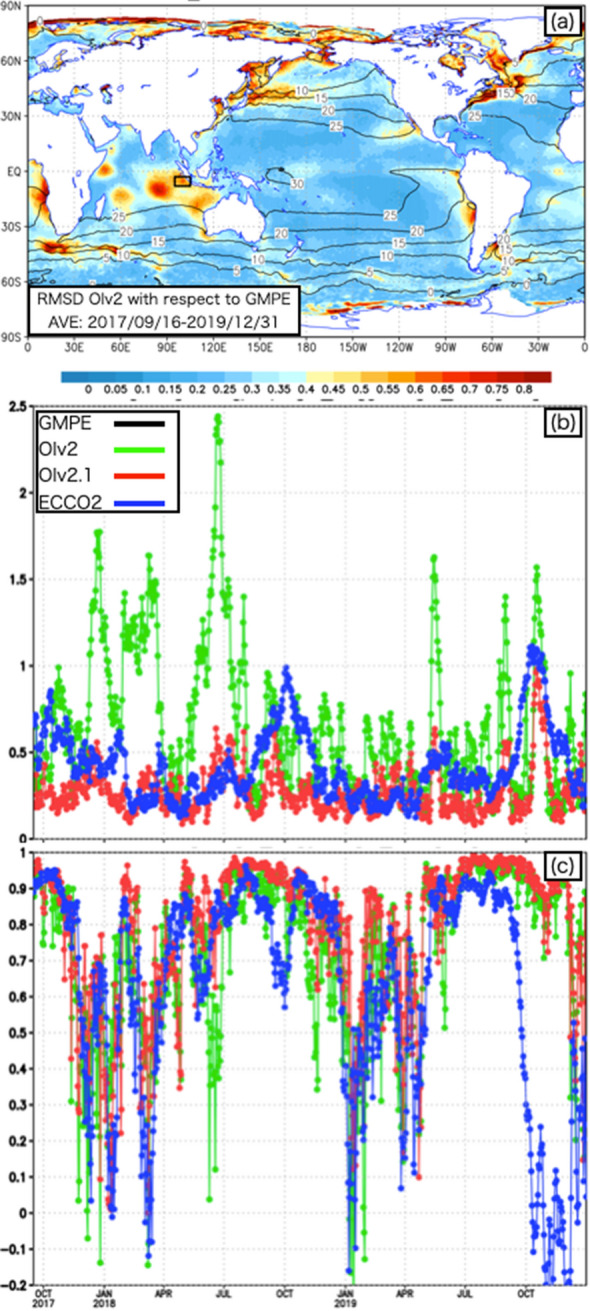
(**a**) RMSD of OIv2 with respect to GMPE (color) and SST with GMPE (contour) for the period from 1 Dec. 2017–31 Dec. 2019. Time series of area-averaged (**b**) RMSD and (**c**) the spatial correlation (SC) with respect to GMPE for the period from 16 Sep. 2017–31 Dec. 2019. OIv2, OIv2.1, and ECCO2 are shown in green, red, and blue, respectively. The averaged area is shown by the black box in (a).

Figure 2a shows the RMSD distribution of OIv2 with respect to GMPE averaged for the period of 16 Sep. 2017–31 Dec. 2019. The large RMSD of OIv2 is not localized in the area off the western coast of Sumatra, and the RMSD of 0.5–1 K is widely distributed in the eastern Indian Ocean, especially in the Southern Hemisphere from 0 to 30° S. Focusing on the tropics from 15° S to 15° N, areas with large RMSD are seen in the coastal regions off western Africa, South America, and northern Australia, which could correspond to coastal upwelling regions. Over the Indian Ocean, multiple areas with large RMSD are distributed in the western, central, and eastern parts. These areas with large RMSD would be a significant issue when summarizing various results that use different L4 SST datasets. For example, different SST datasets could lead to different processes, results, and theories, even for the same phenomenon.

Figure 2b shows the time series for the RMSDs of OIv2, OIv2.1, and ECCO2 with respect to GMPE averaged for the area from 3–8° S, 95–105° E, where diurnal convection originating from Sumatra propagates^15^; this region is the main target area for air-sea interaction research related to YMC. The verification target area is selected because the suitability of the L4 SST datasets has a large impact as a premise for various YMC studies. Although OIv2 repeatedly shows peaks that exceed 1 K and last for 1 week–1 month, the RMSD of OIv2.1 is generally less than 0.5 K throughout the entire period. The RMSD of ECCO2 has a peak around Oct. each year associated with the seasonal variation in the large meridional gradient of SST from 3 to 8° S. The RMSD variation in ECCO2 occurs because the position of the large SST gradient in ECCO2 is shifted from that in GMPE.

As for the time series of the spatial correlation (SC) against GMPE (Fig. 2c), all of OIv2, OIv2.1, and ECCO2 show the decrease from Oct. to Jun. in the next year due to the inhomogeneity, and the increase from Jul. to Sep. due to the homogeneity in the targeted area of 3–8° S, 95–105° E. The homogeneity and inhomogeneity of SST in the targeted area are mainly generated in association with the seasonal migration of the local maximum of the meridional SST gradient. The significant decrease of SC with ECCO2 from Oct. to Dec. in 2019 was due to the large shift of the position of the SST gradient maximum from that with GMPE.

### Validation for Dec. 2017 during the period of the MR17-08 cruise

**Figure 3:**
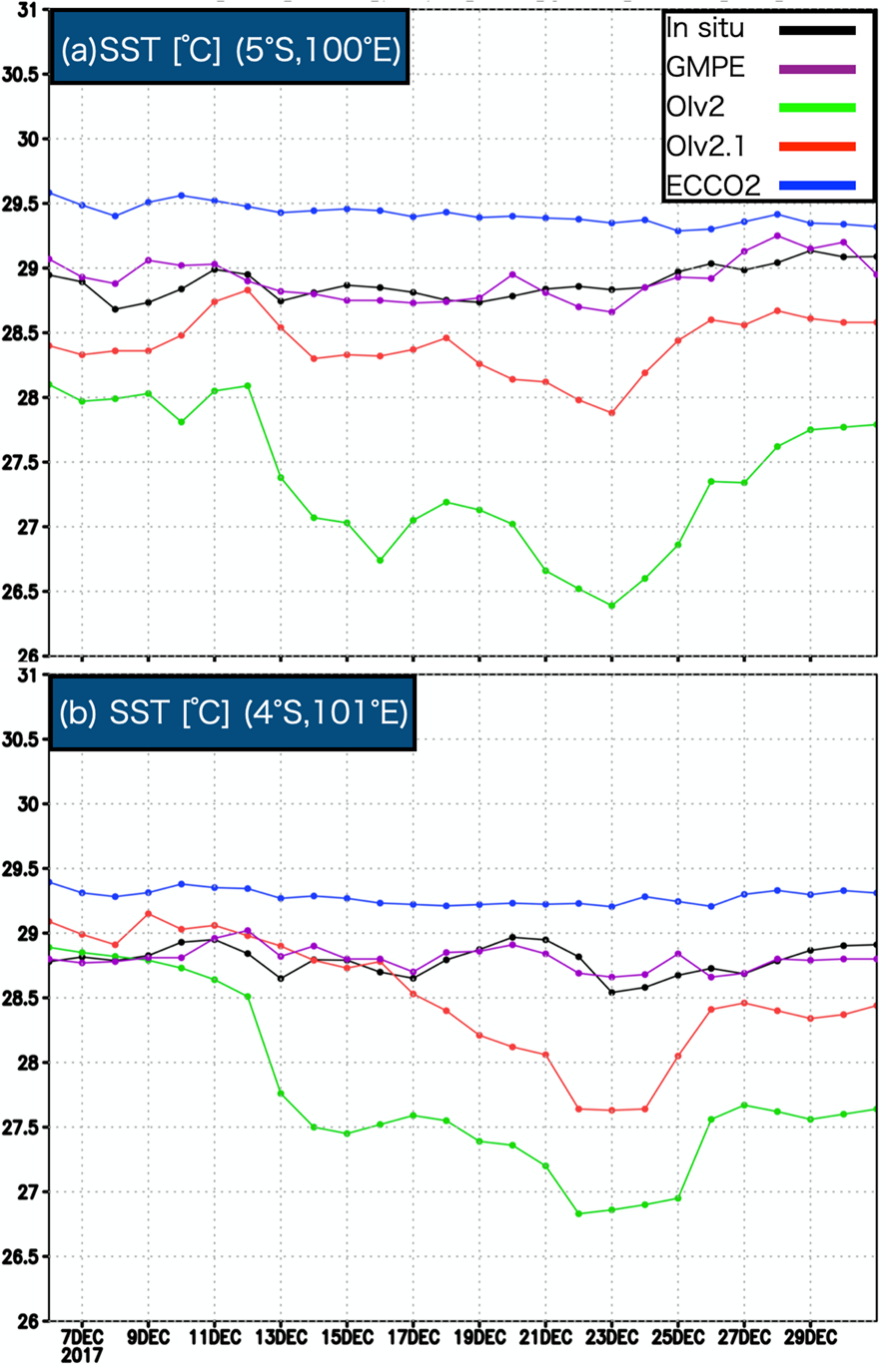
Daily SST variations obtained with respect to (**a**) the Sumatra buoy [5° S, 100° E] and (**b**) R/V Mirai CTD data [4° S, 101° E] (black) from GMPE (purple), OIv2 (green), OIv2.1 (red), and ECCO2 SST (blue).

Because there are a lot of ongoing studies regarding the MJO passage over the Maritime continent and diurnal convection originated from Sumatra during the R/V Mirai cruise period of MR17-08 in the 2017 December, this study considers the factors that caused the difference in SST distribution. Figure 3 shows the time series of SST (a, b) at the Sumatra buoy and in the fixed-point R/V Mirai CTD profiles during the MR17-08 cruise from 6 Dec. to 31 Dec. 2017. The in situ observed SST variation is smaller than approximately 1 K because the mixed layer was well developed (80–120 m in depth). The mixed layer deepening could have been due to the strong westerly winds (5–10 m/s) that were dominant after the MJO passage. OIv2 shows a large negative bias for the entire period of the MR17-08 cruise, and its variation is quite different from that of the in situ SST. OIv2.1 is much improved in terms of temporal variations, although it still has a negative bias with respect to the Sumatra buoy. In addition, OIv2.1 still has some negative bias after 17 Dec., although it is very consistent with the R/V Mirai CTD data before 17 Dec. ECCO2 shows a positive bias, and its variation amplitude is smaller than that of the in situ observations.

**Figure 4:**
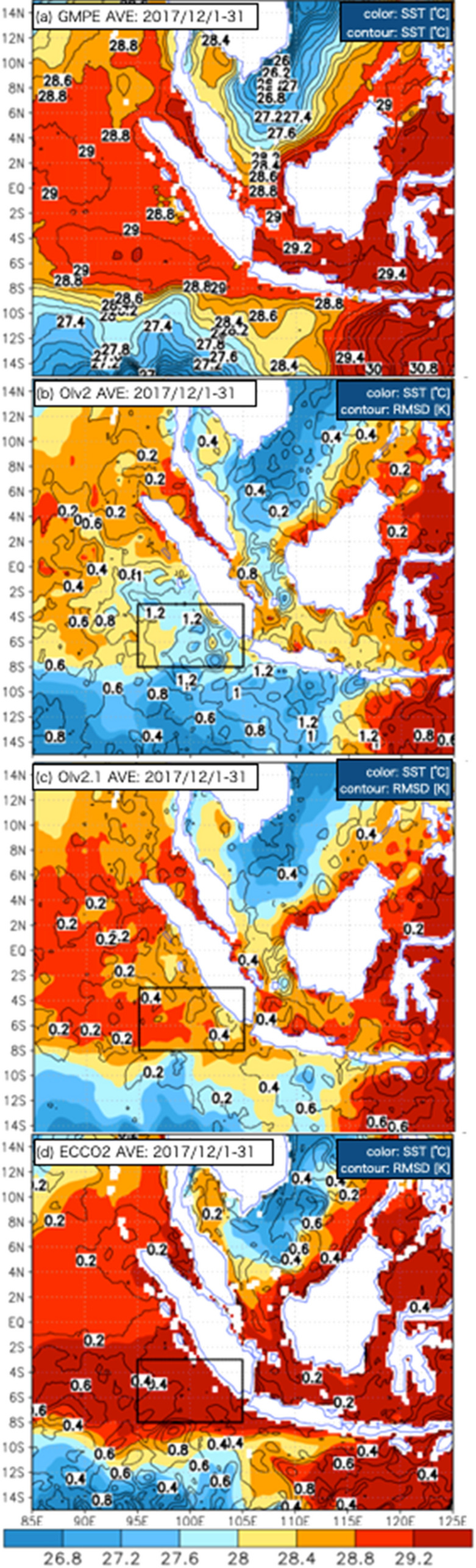
(**a**) GMPE (color and contours every 0.2 K), (**b**) OIv2, (**c**) OIv2.1, and (**d**) ECCO2 for 1–31 Dec. 2017. The RMSD with respect to GMPE is indicated with contours every 0.2 K in (**b**–**d**). Spatial correlation and regression values with respect to GMPE calculated for the box from 3–8° S 95–105° E are shown at the top right corner of each panel in (**b**)-(**d**).

Figure 4 shows the average SST distributions of GMPE, OIv2, OIv2.1, and ECCO2 for Dec. 2017. During this period, which includes the MR17-08 cruise, there are remarkable differences in the SST distributions in the area off the western coast of Sumatra in the Southern Hemisphere. In the OIv2 distribution (Fig. 4b), low SSTs less than 28 °C, which are clearly different from those of GMPE, are distributed off the western coast of Sumatra and extend to the equator from the south. The large RMSDs of OIv2 exceeding 1 K are distributed in the area off the western coast of Sumatra, the southern coast of Java, and the inland sea between the islands of Borneo and Java. As mentioned on the website providing OIv2 and OIv2.1^16^, the quality of the satellite data used in OIv2 may be degraded in such areas, for example, due to the continuous existence of deep convection. In the distribution of OIv2.1 (Fig. 4c), the unrealistically low SST in OIv2 is corrected in many areas, although the RMSD of approximately 0.4 K due to the unrealistically low SST off the western coast of Sumatra partly remains. Over the open ocean of the Indian Ocean, the RMSD of OIv2.1 is generally less than 0.2 K, and OIv2.1 basically presents a distribution that is very similar to that of GMPE. Focusing on the SST front, as indicated by the contours of 28.4–28.8 °C, the distribution of ECCO2 (Fig. 4d) is more similar to that of GMPE than to that of OIv2, although a positive bias with respect to GMPE is seen in the vicinity of the MC. Although Moteki et al.^4^ showed that ECCO2 data has a positive bias off the coast of western Sumatra, we found here that the explanation for several large RMSDs of 0.4–1.2 K with positive bias is the difference between the magnitude of the meridional SST gradient from 6 to 10° S and that of GMPE.

**Figure 5:**
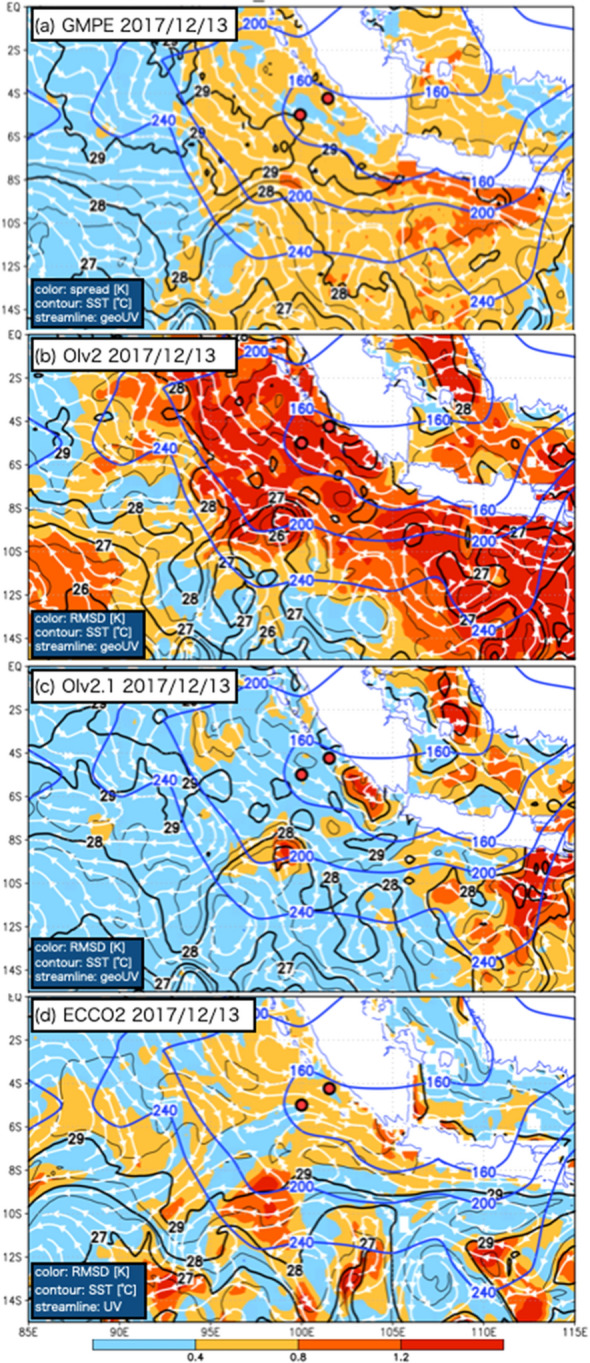
The estimated error (colored) as the (**a**) GMPE ensemble standard deviation, (**b**) RMSD of OIv2 with respect to GMPE, (**c**) RMSD of OIv2.1 with respect to GMPE, and (**d**) RMSD of ECCO2 with respect to GMPE on 13 Dec. 2017. The black contours (every 0.5 K is indicated with thin contours, and every 1 K is indicated with bold contours) show SST from (**a**) GMPE, (**b**) OIv2, (**c**) OIv2.1, and (**d**) ECCO2. The white streamlines indicate the surface current from (**a**)–(**c**) the geostrophic current calculated from SSH and (**d**) ECCO2. The blue contours show 160, 200, and 240 W/m^2^ OLR. The two red circles indicate the locations of the Sumatra buoy and R/V Mirai CTD.

Figure 5 depicts SST, sea surface height (SSH), the sea surface geostrophic current calculated from SSH, and outgoing longwave radiation (OLR) on 13 Dec. 2017. The area with large RMSDs exceeding 1 K corresponds well to OLRs less than 240 W/m^2^ (indication of continuous deep convection). This fact suggests that a large-scale convective system developing continuously over the MC is one of the factors contributing to the quality degradation of OIv2, as indicated by its large RMSD with respect to GMPE. OIv2 can be expected to degrade away from the immediate vicinity of in situ observations during the period of persistent cloud cover because satellite data used in OIv2 could be restricted or degraded^17,18^. Note that the RMSD of OIv2 does not have a simple proportional relationship with OLRs; thus, their variations do not correlate with each other because the daily updated dataset is corrected by in situ observations. The ensemble spread of the 16 datasets used in GMPE shows that the estimated uncertainties of 0.3–0.5 K, which are larger than those of globally averaged values shown in previous reports (e.g.,^12,13^), are distributed in the area to the north of 6° S off the western coast of Sumatra, although large estimated uncertainties exceeding 0.5 K are found in the area to the south of 6° S, 95–115° E. The large RMSD of OIv2 in the area off the western coast of Sumatra is considered to be caused by inherent problems in the OIv2 estimation process.

OIv2.1 is significantly improved from OIv2 over the whole tropical eastern Indian Ocean. There are several large RMSDs in OIv2.1 exceeding 1 K in the areas off the southern edge of Sumatra (5–8° S, 102–105° E), along the eastern edge of the eddy off the southern coast of Java (10–15° S, 105–110° E), and in the inland sea (0–4° S, 107–109° E). The improvements of OIv2.1 from OIv2 could be due to the replacement of satellite inputs to remove degraded satellite data^17,18^ because the RMSDs of OIv2.1 are clearly decreased from that of OIv2 even in the area with low OLRs (less than 240 W/m^2^).

Because the SST of ECCO2 is estimated after dynamically considering the ECCO2-estimated ocean current, the estimated uncertainty of the ECCO2 ocean current is one of the main factors causing the ECCO2 SST uncertainty. There are some shifts in position and size between the eddies obtained with ECCO2 and observed SSH to the south of 10° S, and these shifts are considered to be a factor causing the large RMSD of ECCO2 SST in the vicinity of the eddies.

## Discussion

This study verifies the L4 SST datasets, including GMPE, in the area off the western coast of Sumatra against in situ water temperature values obtained from the Sumatra buoy (5°S, 100° E) and CTD profiles operated by the R/V Mirai (fixed at 4.24° S, 101.52° E). GMPE is confirmed to be the most reasonable L4 SST according to the validation against the reference of in situ observations. However, GMPE has the disadvantages of providing a short dataset period (from 16 Sep. 2017 to present) and no SST anomaly values. Because the datasets of OIv2, OIv2.1, and ECCO2, which are stably operated over long term, are reasonable candidates to compensate the disadvantages of GMPE, they are statistically validated with respect to the GMPE reference.

Analyzing the time series for 2017–2019 off the western coast of Sumatra, OIv2 continuously showed a significant underestimation through the years and its variation was often different from GMPE. OIv2.1 showed a reduction in the negative bias of OIv2 and its variation was well consistent with GMPE. ECCO2 was in good agreement with GMPE for increasing SST variations from Feb. to Aug., although it tended to be overestimated from Sep. to Jan.

Focusing on the period of Dec. in 2017 for which there are a lot of ongoing YMC studies, OIv2 has not only large negative bias but also unnatural variation which is not seen in in-situ observations, GMPE, OIv2.1, and ECCO2. The RMSD of OIv2 was found to be drastically increased after 13 Dec. in 2017 in association with the MJO clouds.

Among the RMSD results of the OIv2 distribution with respect to the GMPE reference, large RMSDs were found to be widely distributed across the eastern Indian Ocean rather than representing a local feature off the western coast of Sumatra. Multiple areas with large OIv2 RMSD (0.5–1 K) were distributed in the western, central and eastern regions of the Indian Ocean. These large RMSDs indicate a serious problem that can affect interpretations when conducting various analyses and numerical experiments related to YMC. Atmospheric simulation results can vary significantly depending on the choice of SST dataset.

In areas of the Northern Hemisphere and to the east of 115° E, the monthly averaged SST distributions of the 4 SST datasets were generally similar to each other. However, there were significant differences between the datasets in the areas off the western coast of Sumatra, the southern coast of Java, and the Indonesian inland sea. This feature was consistent with the ensemble spread distribution from GMPE. Although low SSTs less than 28 °C, which were clearly different from those of GMPE, were located off the western coast of Sumatra and extended to the equator from the south in OIv2, this distribution was improved in OIv2.1. The area with large RMSDs corresponded well to the continuous occurrence of deep convection. Changes in the satellite data used for estimation were suggested to have contributed to the improvement in OIv2.1. Another factor contributing to the improvements in OIv2.1 would be increases in the number of in situ observations. However, considering the horizontal correlation length scales of OIv2 and OIv2.1 of 150–200 km, the wide area with large RMSDs with the large scale more than 1000 km would be mainly due to the change in satellite data.

## Summary

In summary, YMC, a multiyear (from 2015 to present) international program with participants from more than 15 countries, is being conducted. Understanding the characteristics of and differences in SST datasets in the vicinity of the MC is very important as a premise for discussion. This study validated the L4 SST datasets of GMPE, OIv2, OIv2.1, and ECCO2 in the area off the western coast of Sumatra against in situ observations.

GMPE is the most reasonable L4 SST dataset according to validation with Argo floats worldwide. GMPE shows very small RMSD < 0.2 K according to validation with the Sumatra buoy and R/V Mirai CTD data obtained during the MR17-08 cruise, even in the area near the western coast of Sumatra. Although the RMSD of OIv2 off the western coast of Sumatra is very large (1–1.5 K) and associated with a significant negative bias, that of OIv2.1 is improved (RMSD < 0.4 K) in many areas. The RMSD of ECCO2 is less than 0.4 K and associated with a positive bias. Notably, ECCO2 shows a slightly higher TC than OIv2.1.

## Methods

### In situ observations

In this study, the following two in situ observation stations in the marginal sea off the western coast of Sumatra were used for validation. The Sumatra buoy was deployed at 5° S, 100° E (approximately 270 km from the western coast of Sumatra) as a mission of the R/V Mirai MR17-08 cruise. Hourly water temperature profiles were obtained from 5 Dec. 2017 to 10 Dec. 2018 (observations stopped due to communication trouble).

During the R/V Mirai MR17-08 cruise period of 6 Dec. 2017–1 Jan. 2018, CTD observations were conducted 8 times per day, every 3 h, at the fixed point of 4.24° S, 101.52° E (approximately 100 km from the western coast of Sumatra). Here, we use the 1-day average water temperature from Sumatra buoy and the CTD profiles at 10 m depth as a reference for SST. As a result of examining the different depths between 1 and 10 m, the values of each index shown in this study (RMSD, TC, RC, and variance) do not change much.

For the observational errors of the Sumatra buoy and CTD, the expected measurement error is ± 0.001 °C according to the manufacturing datasheet^19^. The time representation uncertainty within 24 h estimated from the variance of fluctuations is estimated to be 0.03 K on average and 0.28 K at maximum. Although the spatial representation uncertainty could vary depending on the current velocity, the maximum travel distance for 24 h assuming 0.3 m/s is approximately 26 km (0.3 m/s × 24 h × 3600 s), which almost corresponds to the horizontal size of the 1/4° grid. The estimated horizontal representation uncertainty of the targeted daily SST products with a 1/4° grid is 0.03 K on average and 0.28 K at maximum.

### Validated target SST products

Although there are many L4 SST datasets, we chose the following 4 widely used datasets with a spatial resolution of 0.25° and daily temporal resolution to be validated with the YMC in situ SST observations. GMPE is the ensemble median of 16 L4 SST datasets provided by the Copernicus Marine Environment Monitoring Service (CMEMS) for the period from 16 Sep. 2017 to present^13^. OIv2, including data from 1 Sep. 1981 to 26 Apr. 2020^20^, is one of the most popular daily L4 SST datasets and has been widely used in many studies. OIv2.1 was released as an update to OIv2; data are available from 1 Sep. 1981 onward^16,17^. Major improvements from OIv2 to OIv2.1 include (1) large increases in the number of in situ ships, buoys, and Argo floats used and (2) the change of satellite inputs from METOP-A and NOAA-19 to METOP-A and METOP-B to remove degraded satellite data^17,18^. The ECCO2 dataset has 50 vertical levels at a maximum model depth of 6150 m with a high global ocean resolution of 0.25° × 0.25°^21,22^. ECCO2 is an adjoint method-based state estimation constrained to the available satellite (SST and SSH) and in situ (vertical temperature and salinity profiles) data, is available from 16 Sep. 1992, and is updated with a few months’ delay. Note that the difference in the correlation length scales between datasets could affect the representation of the SST distribution. The horizontal correlation length scales of OIv2, OIv2.1, and ECCO2 would be approximately 150–200 km, although the correlation length scale of the GMPE system could be approximately 100 km in the tropics (e.g.,^23^).

The sea surface geostrophic current calculated from the SSH of the Data Unification and Altimeter Combination System (DUACS) multimission gridded (L4) altimeter products with a spatial resolution of 0.25° and daily temporal resolution^24,25^ was used. In addition, the daily estimates of OLR with a 2.5° resolution from polar-orbiting satellites^26^ were provided by NOAA to confirm convective activities.

## References

[1] Yoneyama K, Zhang C (2020). Years of the maritime continent. Geophys. Res. Lett..

[2] Kinoshita T (2019). A study of gravity wave activities based on intensive radiosonde observations at Bengkulu during YMC-Sumatra 2017. IOP Conf. Ser. Earth Environ. Sci..

[3] Katsumata M, Taniguchi K, Nishizawa T (2020). An attempt to retrieve continuous water vapor profiles in marine lower troposphere using shipboard Raman/Mie lidar system. SOLA.

[4] Moteki Q, Katsumata M, Yoneyama K, Ando K, Hasegawa T (2018). Drastic thickening of the barrier layer off the western coast of Sumatra due to the Madden-Julian oscillation passage during the pre-years of the maritime continent campaign. Prog. Earth Planet. Sci..

[5] Yokoi S (2017). Diurnal cycle of precipitation observed in the western coastal area of Sumatra Island: offshore preconditioning by gravity waves. Mon. Weather Rev..

[6] Yokoi S, Mori S, Syamsudin F, Haryoko U, Geng B (2019). Environmental conditions for nighttime offshore migration of precipitation area as revealed by *in situ* observation off Sumatra Island. Mon. Weather Rev..

[7] Yokoi S (2020). Diurnal variation of surface heat fluxes off the west coast of Sumatra Island as revealed by *in situ* observation. SOLA.

[8] Wu P, Ardiansyah D, Mori S, Yoneyama K (2019). The effect of an active phase of the Madden-Julian oscillation on surface winds over the western coast of Sumatra Island. IOP Conf. Ser. Earth Environ. Sci..

[9] Zhao N, Nasuno T (2020). How does the air-sea coupling frequency affect convection during the MJO passage?. J. Adv. Model. Earth Syst..

[10] Madden RA, Julian PR (1972). Description of global-scale circulation cells in the tropics with a 40–50 day period. J. Atmos. Sci..

[11] Wang B, Xie X (1997). A model for the boreal summer intraseasonal oscillation. J. Atmos. Sci..

[12] Dash P, Ignatov A, Kihai Y, Sapper J (2010). The SST quality monitor (SQUAM). J. Atmos. Ocean. Technol..

[13] Martin M (2012). Group for high resolution sea surface temperature (GHRSST) analysis fields inter-comparisons. Part 1: A GHRSST multi-product ensemble (GMPE). Deep Sea Res. II Top. Stud. Oceanogr..

[14] JAMSTEC & BPPT. *MR17-08 Cruise Report* http://www.godac.jamstec.go.jp/catalog/data/doc_catalog/media/MR17-08_leg1-2_all.pdf (2018).

[15] Mori S (2004). Diurnal land–sea rainfall peak migration over Sumatera Island, Indonesian maritime continent, observed by TRMM satellite and intensive rawinsonde soundings. Mon. Weather Rev..

[16] NOAA. *Optimum Interpolation Sea Surface Temperature (OISST) v2.1* https://www.ncdc.noaa.gov/oisst/optimum-interpolation-sea-surface-temperature-oisst-v21 (2020).

[17] Huang B (2021). Improvements of the daily optimum interpolation sea surface temperature (DOISST) version 2.1. J. Clim..

[18] Banzon V, Smith TM, Steele M, Huang B, Zhang HM (2020). Improved estimation of proxy sea surface temperature in the Arctic. J. Atmos. Ocean. Technol..

[19] Sea Bird Scientific. *SBE 911plus CTD* https://www.seabird.com/profiling/sbe-911plus-ctd/family-downloads?productCategoryId=54627473769 (2021).

[20] Banzon V, Smith TM, Chin TM, Liu C, Hankins W (2016). A long-term record of blended satellite and *in situ* sea-surface temperature for climate monitoring, modeling and environmental studies. Earth Syst. Sci. Data.

[21] Halpern D, Menemenlis D, Wang X (2015). Impact of data assimilation on ECCO2 equatorial undercurrent and north equatorial countercurrent in the Pacific ocean. J. Atmos. Ocean. Technol..

[22] Menemenlis, D. *et al. ECCO2: High Resolution Global Ocean and Sea Ice Data Synthesis* https://www.mercator-ocean.fr/wp-content/uploads/2015/06/lettre_31_en.pdf#page=13 (2008).

[23] De Meyer V, Roca R (2021). Thermodynamic scaling of extreme daily precipitation over the tropical ocean from satellite observationsthermodynamic scaling of extreme daily precipitation over the tropical ocean from satellite observations. J. Meteorol. Soc. Jpn..

[24] Pujol I (2016). DUACS DT2014: the new multi-mission altimeter data set reprocessed over 20 years. Ocean Sci..

[25] Pujol MI (2018). Gauging the improvement of recent mean sea surface models: a new approach for identifying and quantifying their errors. J. Geophys. Res. Oceans.

[26] Gruber A, Krueger AF (1984). The status of the NOAA outgoing longwave radiation data set. Bull. Am. Meteorol. Soc..

